# Nanoporous CuS with excellent photocatalytic property

**DOI:** 10.1038/srep18125

**Published:** 2015-12-09

**Authors:** Wence Xu, Shengli Zhu, Yanqin Liang, Zhaoyang Li, Zhenduo Cui, Xianjin Yang, Akihisa Inoue

**Affiliations:** 1 School of Materials Science and Engineering, Tianjin University, Tianjin, 300072, China; 2 Tianjin Key Laboratory of Composite and Functional Materials, Tianjin, 300072, China; 3 Key Laboratory of Advanced Ceramics and Machining Technology, Ministry of Education, Tianjin, 300072, China

## Abstract

We present the rational synthesis of nanoporous CuS for the first time by chemical dealloying method. The morphologies of the CuS catalysts are controlled by the composition of the original amorphous alloys. Nanoporous Cu_2_S is firstly formed during the chemical dealloying process, and then the Cu_2_S transforms into CuS. The nanoporous CuS exhibits excellent photocatalytic activity for the degradation of the methylene blue (MB), methyl orange (MO) and rhodamine B (RhB). The excellent photocatalytic activity of the nanoporous CuS is mainly attributed to the large specific surface area, high adsorbing capacity of dyes and low recombination of the photo generated electrons and holes. In the photo degradation process, both chemical and photo generated hydroxyl radicals are generated. The hydroxyl radicals are favor in the oxidation of the dye molecules. The present modified dealloying method may be extended for the preparation of other porous metal sulfide nanostructures.

One of the increasingly severe challenges in chemistry is diminishing the detrimental environmental impact associated with chemical industries. Advanced oxidation process (AOP) is an efficient way to solve pollution problems for different reacting systems by producing oxidizing OH**·** radicals via Fenton, photo-Fenton reactions and photocatalysis. The versatility of AOP makes it very flexible to offer OH**·** radicals for the specific treatment requirements^1^. Sunlight, a pollution-free and easily available energy resource, possesses great potential in driving environmentally benign organic transformations^2^. Especially for the AOP, the introduction of light is necessary for the photocatalysis and enhances dramatically the activity of Fenton reactions. As the most famous photocatalyst, TiO_2_ has been widely studied since Fujishima first reported the photoelectrochemical water-splitting on the TiO_2_ electrode^3^. However, the large energy gap makes TiO_2_ can solely absorb the UV light, thus greatly depress the generation of hydroxyl radical which plays a key role in the oxidative destruction of organic pollutant. Hence, it is highly desirable to develop photocatalysts with high generation rate of hydroxyl radicals. To achieve this goal, numerous novel single-phase semiconductors with narrow bandgap have been developed to enhance the photocatalytic efficiency, such as graphitic-C_3_N_4_ and Ag_3_PO_4_^4^,^5^. On the other hand, the efficient of photo-Fenton reactions is dependent on the pH value, It was reported that the optimum pH value for photo-Fenton reaction is 2.8 because under this pH the precipitation could be totally dissolved and further promote the formation of active [Fe(OH)]^2+^ in water. However, it is uneconomical to operate photo-Fenton reactions at this pH value since it requires high chemical costs for pH rectification^6^. Hence, new types catalysts are urgently needed to overcome these drawbacks of photocalysis and photo-Fenton reactions.

Metal sulfides have attracted extensive attention in the recent years. Among these materials, copper chalcogenides exhibit unique optical, electronic, physical and chemical properties with prospective and numerous applications as both the photocatalysis and the Fenton-like reactant^7^,^8^,^9^,^10^,^11^,^12^,^13^. In addition, the CuS introduced Fenton-like reactions is free from iron ions, leading to the separation of reactants from ions to solid materials. The degradation of organic dye molecules in the CuS introduced Fenton-like reactions can be proceed efficiently without the adjustment of pH value. These advantages make the CuS introduced Fenton-like reactions more cost efficient by avoiding the ion removal and pH rectification. On the other hand, the elements S and Cu in the copper chalcogenides are “liquidus” so as to fabricate other materials without destruction of morphology^14^. In order to obtain efficient charge separation and transportation, many copper chalcogenide nanostructures, such as nanospheres, nanodisks, nanocrystals and nanowires were designed with short charge diffusion length, high crystallinity and less defects^15^,^16^,^17^,^18^. Among these materials, porous nanostructure draws intensive interests because the nanoporous structure not only processes large active surface area but also prevents agglomeration of catalysts^19^. However, the efficient synthesis of nanoporous copper chalcogenides still remains a challenge.

Dealloying is a traditional method to fabricate porous structures. Dealloying is a method that less stable elements in the alloy are dissolved out to leave a nanoporous film, skeletal surface or core-shell particle configuration overlying a composition closer to a stable bulk alloy composition^20^. The composition and pore size can be easily tuned by adjusting the constituent of the original alloy. However, only pure metal or metal alloy porous structures can be prepared by conventional dealloying^21^. Nanoporous metal sulfides fabricated by dealloying method have not been reported before.

Here we report a facile dealloying strategy to fabricate nanoporous copper chalcogenides for the first time. The evolution of the copper chalcogenide nanoporous structure is described as well. The as-prepared porous CuS nanostructure exhibits unique light absorption performance and high photocatalytic activity towards photodegradation of methylene blue (MB), methyl orange (MO) and Rhodamine B (RhB).

## Results and Discussion

**Figure 1:**
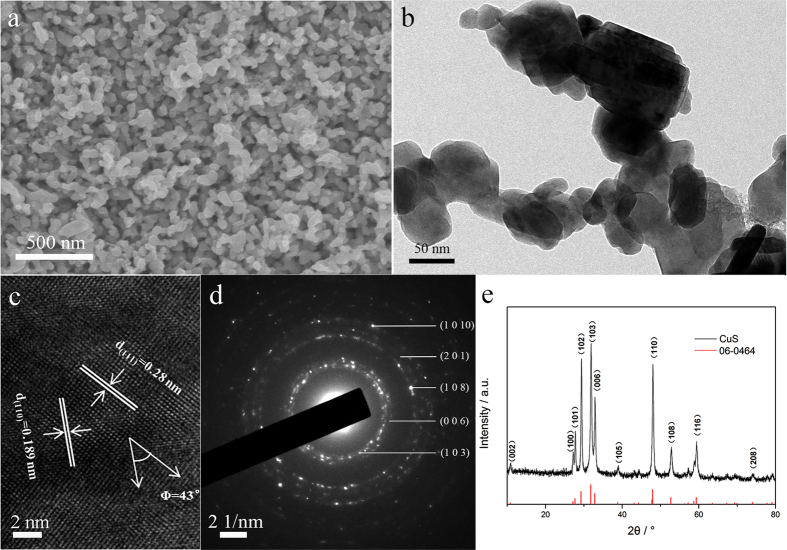
SEM image (**a**), TEM image (**b**), HRTEM image (**c**), diffraction pattern (**d**) and XRD pattern (**e**) of the nanoporous CuS.

Typical SEM image of the as-formed nanoporous CuS (np-CuS) is shown in Fig. 1(a). This material consists of a uniform bicontinuous network with sizes of both the ligaments and pores of about 50 nm. The np-CuS exhibits a homogeneously connected structure from outside to inside which is favor of transmission of reactants. Figure 1(b) shows the TEM image of the np-CuS. The ligaments of the np-CuS are composed of a number of nanoparticles. A HRTEM image was taken to further examine the crystallographic features of np-CuS. According to Fig. 1(c), lattice distances of the sample can be determined as 0.19 ± 0.01 nm and 0.28 ± 0.01 nm, which correspond to the (110) and (111) plane of covellite, respectively. The angle between (110) and (111) planes was 43° which corresponds to the theoretical value of interfacial angle between the (110) and (111) planes. Figure 1(d) shows the electron diffraction (ED) pattern of the np-CuS sample. Only a single set of diffraction rings appears in the pattern which can be indexed as covellite phase. This result is coincided with the HRTEM image. Figure 1(e) is the XRD pattern of the as-prepared sample. All diffraction peaks agree with the covellite phase (JCPDS No.06-0464). As confirmed by X-ray diffraction (XRD), the np-structure was identified with the pure CuS, indicating that the Ti-Cu amorphous alloy completely transformed into crystalline CuS.

**Figure 2:**
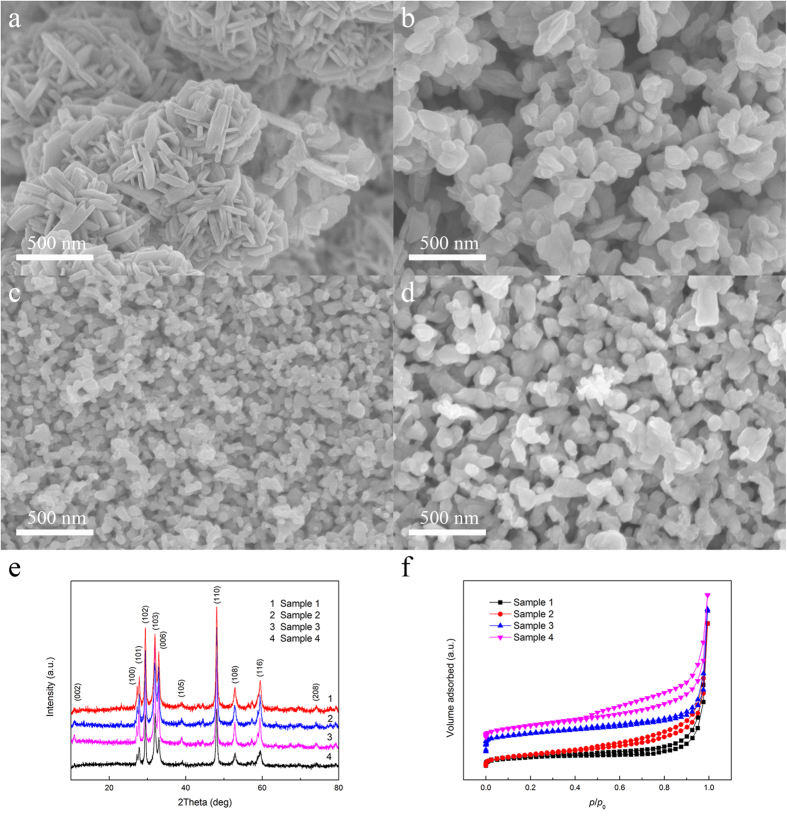
SEM images of the CuS catalysts prepared by different Ti-Cu amorphous alloys, (**a**) Sample 1, (**b**) Sample 2, (**c**) Sample 3 and (**d**) Sample 4. (**e**) XRD patterns of the CuS catalysts prepared by different Ti-Cu amorphous alloys. (**f**) N_2_ adsorption–desorption isotherms of the CuS catalysts prepared by different Ti-Cu amorphous alloys.

As shown in Fig. 2, different morphologies were generated for different Ti:Cu ratio of the amorphous alloy. The products prepared by Ti_50_Cu_50_, Ti_40_Cu_60_, Ti_30_Cu_70_ and Ti_20_Cu_80_ were named as Sample 1, 2, 3 and 4, respectively. For the low Cu content (such as Ti_50_Cu_50_), nanosheet-composed globular clusters were formed. The diameter of the cluster is about 500 nm and the thickness of the nanosheet is about 40 nm. With increasing Cu content in the original Ti-Cu amorphous alloy, the nanosheets became smaller and gradually evolved into smooth clusters of nanoparticles (as shown in Fig. 2(b)). With further increasing Cu content, the nanoparticles refined furtherly, and formed bi-continuous porous nanostructure (as shown in Fig. 2(c)). Very high Cu content (such as Ti:Cu ratio of the original amorphous alloy was 20:80) would make the nanoporous structure coarse (as shown in Fig. 2(d)). The formation of the ligaments can be summarized as the following mechanism. The relative stable element (Cu) on the surface formed clusters firstly, then newly exposed alloy would further reacted with the acid to form the continuous ligaments. The underlying reactive atoms would undergo a dissolution process and inevitably influence the previous atom rearrangement. As a result, the voids created upon the dissolution of these reactive atoms might trigger new atom rearrangements locally, further disturbed the previously ongoing atom rearrangement^22^. Hence, the atomic rearrangement was dominated strongly by the composition of the original amorphous alloy. When the content of Cu is low, the dissolution of the active atoms dominates the dealloying process, and the rearrangements are suppressed. With further increasing Cu content, more Cu atoms can participate in the rearrangement process. On the other hand, with an increase of Cu in the original amorphous alloy, the content of Ti decreases correspondingly, and the interspace generated by the dissolution of Ti decreases. When the Cu content exceeds a certain value, the Cu clusters would connect with each other, and hence the continuous ligaments are formed. When the Cu content increases up to 80%, the rearrangement of Cu is dominant, resulting in the increased size of Cu ligaments.

The composition of the original amorphous alloy plays a crucial role in the creation of the nanoporous copper chalcogenides. X-ray diffraction analysis (Fig. 2e) suggests that all the samples prepared in 15 M H_2_SO_4_ would possess the same diffraction peaks and be indexed to the pure covellite phase. The XRD patterns indicate that the composition of the original amorphous alloys affects little on the phase of the products.

To futher understand the influence of the composition of the original amorphous alloy on the specific surface area, N_2_ adsorption–desorption isotherms of these CuS cantalysts were investigated. As shown in Fig. 2(f), the specific surface area of the Samples 1, 2, 3 and 4 are 9.324, 21.620, 28.745 and 4.284 m^2^ g^−1^, respectively. Nanoporous CuS prepared by Ti_30_Cu_70_ alloy shows the largest specific surface area. Normally the catalyst with high specific surface area exhibits high photocatalytic activity. Combined with the SEM images, the CuS prepared by the Ti_30_Cu_70_ amorphous alloy is an ideal CuS catalyst under the present synthesis conditions.

**Figure 3:**
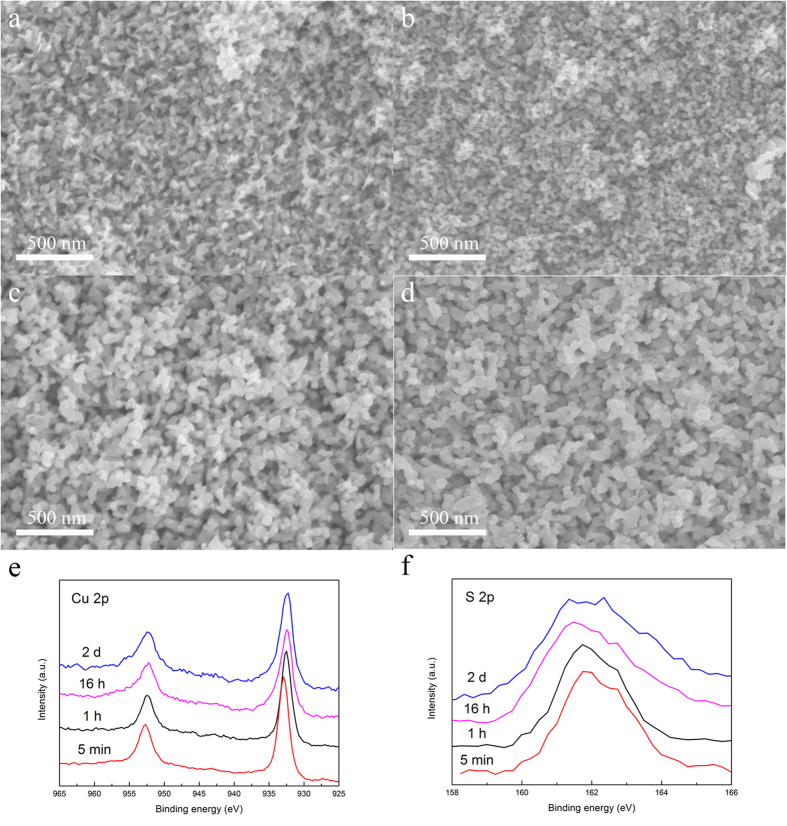
SEM images of the nanoporous CuS prepared by Ti_30_Cu_70_ amorphous alloy for 5 min (**a**), 1 h (**b**), 16 h (**c**), 2 d (**d**) and corresponding XPS spectra of the nanoporous CuS prepared for Cu 2p (**e**) and S 2p (**f**).

Figure 3 shows the morphologies of the Sample 3 during the dealloying process. At the beginning of the reaction, small particles were formed in only tiny fluctuations state on the alloy surface, which was attributed to the etching of the amorphous alloy. Ti was dissolved into the H_2_SO_4_ solution and Cu was transformed into nanoparticles by atoms rearrangement. With further increasing dealloying time, more Ti atoms dissolved into the solution, and the as-formed nanoparticles are connected together. After immersing for 16 h, bicontinuous nanostructure was formed on the surface. At this time the amorphous ribbon was not corroded totally. It is worth to notice that the size of the ligaments was larger than the nanoparticles, which could be attributed to the Ostwald ripening^23^. Finally after 2 days immersion, the ribbon was etched completely, and the nanoporous CuS in the whole ribbon was formed.

XPS patterns of the sample 3 are shown in Fig. 3(e,f) and the fitted curve of S is shown in Fig. S1. Until now the valence of Cu in covellite is still controversial. Some studies suggested a (Cu^1+^)_3_(S_2_^2−^)(S^1−^) or (Cu^1+^)_3_(S_2_^−^)(S^2−^) valence formalism for CuS where the valence of S is equal to −1^24^. Other studies indicate that both Cu^1+^ and Cu^2+^ exist in the covellite CuS^25^. In our case, it can be seen that the peaks located at 932.9 eV and 952.7 eV are corresponding to the Cu 2p3/2 and Cu 2p1/2 of the Cu_2_S, respectively. This indicates that the original nanoporous structure would be Cu_2_S. As the dealloying time increases, the Cu 2p peaks shift negatively in the first hour. This phenomenon can be attributed to the transformation from Cu_2_S to CuS. More obvious changes can be seen in the S 2p region during the evolution from Cu_2_S to CuS. The peaks located at binding energies of 161.7 and 162.8 can be attributed to the S-Cu bonds (the sulfide peak) and the peak located at 163.6 eV corresponds to the S-S dimers (the disulfide moiety)^26^,^27^. At the beginnig of the dealloying process, only S-Cu bonds were detected. This indicates that only Cu_2_S would be generated. With increasing the time, the disulfide was detected and the position of sulfide peak shifted from 161.7 to 161.3 eV, reflecting the decrease in average Cu-S bond length which is smaller in covellite than in chalcocite^28^. The XPS results indicate that the valence of Cu would be +1 during the whole reaction, while the −1 valence of S gradually evolves to −2 and the S content increase gradullay in chemometrics. The total reactions can be described as follows:


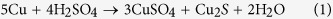



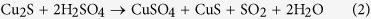


**Figure 4:**
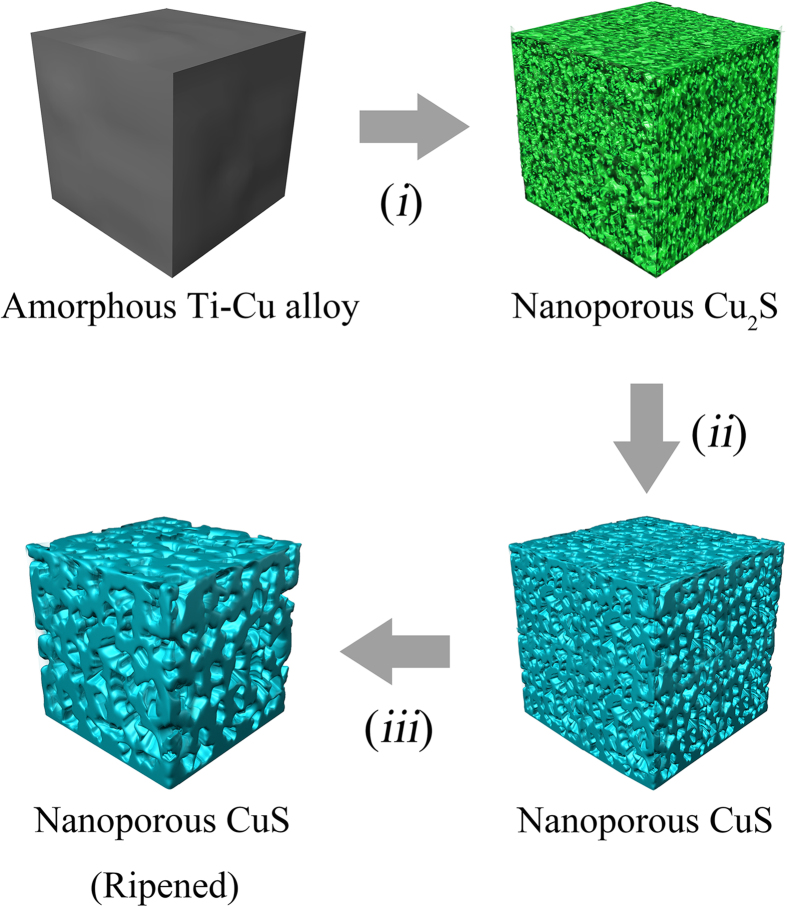
The growth mechanism of the nanoporous CuS. (***i***) Formation of the porous Cu_2_S nanostructure. (***ii***) Sulfurization of the Cu_2_S. (***iii***) Ripening of the nanoporous CuS.

According to the SEM images and XPS patterns, the formation of the nanoporous CuS probably involves three steps: (1) formation of the porous Cu_2_S nanostructure; (2) sulfurization of the Cu_2_S; (3) ripening of the np-CuS (as shown in Fig. 4). At the beginning stage, Ti atoms on the surface were dissolved by the H^+^ from H_2_SO_4_ and more noble Cu atoms trended to agglomerate into islands and condensed onto connected clusters by spinodal decomposition^21^. Meanwhile, the H_2_SO_4_ served as the S source and reacted with the exposed np-Cu to form Cu_2_S cluster. After the np-Cu_2_S was formed, the dealloying and sulfidization occurred on the surface of the Ti-Cu amorphous alloy with continuously increasing surface area. As porous Cu_2_S forms, the Cu_2_S gradually transformed into CuS. After that, small CuS ligaments were etched and entered the larger ligaments according to the Ostwald ripening mechanism.

**Figure 5:**
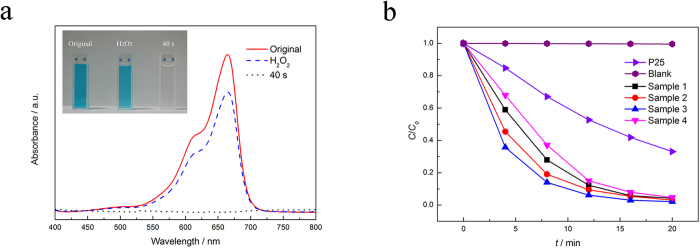
(**a**) Degeneration curves of the 10 mg/L MB solutions. (**b**) The photocatalytic activities of the as-prepared samples for 1000 mg/L MB degradation.

Under irradiation condition, the nanoporous CuS exhibits excellent photocatalytic performance towards the oxidation of the organic dyestuffs without adsorption equilibrium on the surface of the np-CuS. The absorbance curves and real photos of the methylene blue (MB) solution, methyl orange (MO) solution and Rhodamine B (RhB) solution are shown in Fig. 5(a) and Fig. S2. All the dyestuffs were 10 mg L^−1^ in concentration and degraded by the np-CuS with H_2_O_2_ very quickly. Especially, MB was degraded completely within 40 s. This result indicates that the nanoporous CuS is an efficient and universal photocatalyst for photocatalytic degradation of organic dyestuffs.

It is hard to describe accurate degradation curves of these dyes in common test solutions (such as with concentration of 10 mg L^−1^) due to very fast degradation rate. Hence we used a very high concentration MB solution (1000 g L^−1^) in the present degradation test. The results are shown in Fig. 5(b). Commercial P25 was picked as the control group. It is clearly that all the CuS catalysts exhibit excellent photocatlytic performance towards MB oxidation. For all samples, over 95% of MB were decomposed after 20 min, suggesting significant photolytic efficiency. With increasing Cu content in the original amorphous alloy, the photocatalytic activities of CuS catalysts increase firstly. Especially for the Sample 3, the MB decreases to 98% after 16 min. However, when Cu content is over 80% the catalytic activity of CuS decreases. Even though, all the CuS catalysts exhibit obviously higher catalytic performance than the P25. To further characterize the catalytic performances of the catalysts, the reaction rates of the MB degradation are calculated. Langmuir–Hinshelwood pseudo-first order kinetic model is an approved model to characterize the reaction rate of the photocatalytic reactions occurring at a solid-liquid interface which is described as follows^12^:


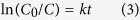


where *k* is the apparent rate constant, *C*_0_ and *C* are the equilibrium concentration of adsorption and the concentration of MB at the relative exposure time, respectively. The plots of the observed first-order rate constants, which are deduced by the slopes of the straight lines in the linear plots of ln(*C*_0_/*C*) versus irradiation time t, are shown in Fig. S3. Table S1 summarizes the calculated apparent rate constant *k*. The values of *k* are 0.181, 0.198, 0.232, 0.161, and 0.056 min^−1^ for Sample 1, 2, 3, 4 and P25, respectively. Hence, the photocatalytic reaction rate of Sample 3 is about 4 times larger than that of P25. In addition, the *k* of the as-prepared CuS catalysts with the presence of H_2_O_2_ is about 50 times higher than that of the other Cu-based catalysts, indicating the excellent photocatalytic property of our np-CuS catalysts.

**Figure 6:**
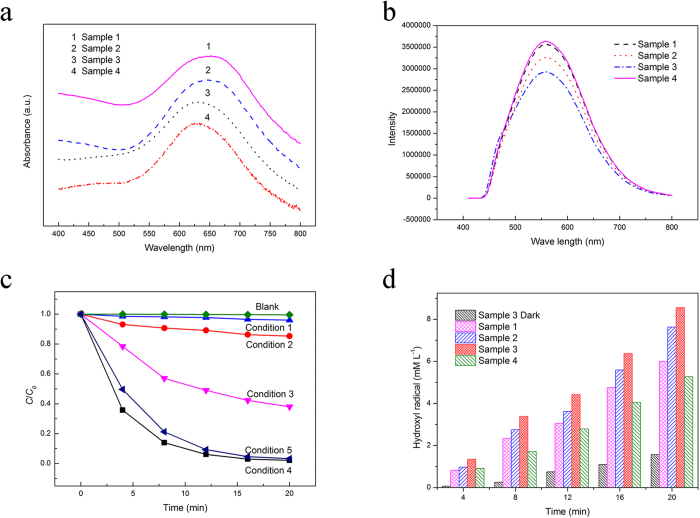
(**a**) UV-vis spectrum of CuS catalysts prepared by different Ti-Cu amorphous alloys. (**b**) Room temperature photoluminescence spectrum of different CuS catalysts. (**c**) Degeneration curves of the nanoporous CuS prepared by Ti_30_Cu_70_ amorphous alloy under different conditions. Blank: pure MB. Condition 1: MB with CuS and dissolved O_2_ under irradiation. Condition 2: MB with H_2_O_2_ and dissolved O_2_ under irradiation. Condition 3: MB with CuS and dissolved O_2_ under dark condition. Condition 4: MB with CuS, H_2_O_2_ and dissolved O_2_ under irradiation. Condition 5: MB with CuS and H_2_O_2_ without dissolved O_2_ under irradiation. (**d**) Total concentrations of HO• formed for CuS catalysts with the assistant H_2_O_2_ as a function of time in saturated BA solution.

The photocatlytic activities of the catalysts depend mainly upon the phase, lattice plane and morphology. The XRD patterns of these CuS catalysts indicates the phases of these sample are similar (as shown in Fig. 2). The light absorption and bandgap energy strongly depends on the phase of the catalysts. Figure 6(a) shows a series of UV-vis absorbance curves for the samples prepared by various Ti-Cu amorphous alloys. All the samples exhibit similar absorbance curves. A significant increase in the absorption at wavelengths shorter than 700 nm can be observed and the bandgap energy are estimated to be 1.6–1.7 eV according to the Kubelka-Munk method (as shown in Fig. S4). The absorbance spectra indicate that the bandgap energy would be only determined by the phase and irrelevant to the morphology. Hence the catalytic performance of nanoporous CuS would be attributed as morphology rather than the phase and lattice plane.

The morphology of the catalyst can strongly affects the mass transfer, adsorption, desorption and recombination of the photo-generated electron/hole pair. The as-prepared CuS catalysts were further characterized by the photoluminescence spectroscopy at room temperature (as shown in Fig. 6(b)). The photoluminescence behavior of CuS has not yet been well understood and always has been explained on the basis of morphology affecting the electronic transition^29^. The peak of the as-prepared CuS is similar and attributed to the similar band gap. However, the intensity of the Sample 3 is the lowest, indicating the lowest recombination rate. Compared to the other morphologies of the as-prepared CuS, the porous nanostructure possesses the largest specific surface area, revealing more trapping sites on the surface of the catalyst. The trapping sites have lower conduction band, and the photogenerated electrons are able to transfer to the trapping sites more easily^30^. Hence the recombination of the photogenerated electrons and holes is reduced. High specific area provided by the nanoporous structure can provide more active sites^31^,^32^. Moreover, the porous nanostructure could promote the material transfer. More MB molecules were adsorbed on the surface of the np-CuS (as shown in Fig. S5). After the dark adsorption process, the adsorption quantity of the np-CuS is 20.47 mg g^−1^ which is the highest among the as-prepared CuS catalysts.

To explore the degeneration mechanism of the np-CuS catalyst, similar degeneration experiments were proceeded in different conditions. As shown in Fig. 6(c), the MB was degenerated barely without CuS and H_2_O_2_, which indicates that MB is stable in the solution. The concentrations of MB in condition 1 and condition 2 changed little after the illumination. This phenomenon indicates that single np-CuS or H_2_O_2_ would exhibit low photocatalytic activity. The photocatalytic mechanism of CuS is similar to the normal photocatalysts such as TiO_2_. The photogenerated holes react with H_2_O to form strong oxidizing OH**·**. Meanwhile, rapid recombination of the photogenerated hole and electron would reduce the photocatalytic activity of CuS. The MB degradation in H_2_O_2_ solution can be explained by the photo-Fenton reactions, i.e. the hemolytic cleavage of H_2_O_2_:


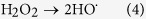


In aqueous solution, the cage effect of water molecules would decrease the primary quantum caused by combination of OH**·** radicals^1^:


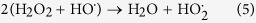



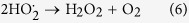


However, it is obvious that even in the dark envirnoment (condition 3), the np-CuS exhibits catalytic activity with the help of H_2_O_2_. As mentioned above, Cu in the CuS possesses +1 state. This makes it possible that Cu^+^ in the CuS reacts with H_2_O_2_ generates hydroxyl radicals, and further leads to a Fenton-like reaction^33^,^34^, which can be deduced as follows^35^:


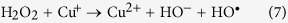



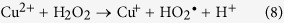


Compared to the dark condition, the degenerate rate of the MB under irradiation (condition 4) is faster than that in condition 3. Moreover, the degenerate rate of MB in condition 4 did not equal to the sum of that in conditions 1, 2 and 3. This is because that the rate of Fenton reactions can be enhanced by the irradiation. Cu^+^ can be regenerated by the photochemical effect. A reversion cycle of Cu^+^ → Cu^2+^ → Cu^+^ continuously generates OH**·**, and further develops the generation rate of the OH**·** radicals in the system^36^. In addition, H_2_O_2_ not only reacts with CuS but also serves as acceptor to attract photogenerated electrons generated by the CuS. The H_2_O_2_ can inhibit the recombination of electron-hole pair, while further provides additional OH**·** radicals through the following mechanisms^6^:


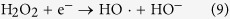



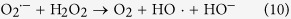


Basu *et al.* reported that the excited electron in CuS can react with dissolved O_2_ to produce active O^2−^ which is active in the photocatalytic process^37^. To detect the effect of the dissolved O_2_ in the phototcatalytic process, photocatalytic test was proceeded under condition 5. Even though the photocatalytic efficiency in condition 5 is a little lower than that in condition 4. However, the photodegradation rate of MB in condition 1 is far less than that in condition 4, indicating the hydroxyl radicals generated from CuS and H_2_O_2_ plays a key role in the photodegradation of the MB. Yu *et al.* suggested that the MB degradation would be a complicated process, which is correlated to the demethylation process and breaking of the central aromatic ring^38^. As the oxidant in MB degradation reactions, the HO• radicals participate in all of these reactions^39^. Hence it is very important to measure the amount of HO• radicals in the photocatalysis process. Figure 6(d) shows the changes in the amount of HO• generated by catalysts as a function of reaction time. The amount of HO• produced by Sample 3 is up to the highest value of about 8.56 mM L^−1^ after 20 min. In addition, even without irradiation, HO• can be also produced in the presence of CuS catalyst with the assistant of H_2_O_2_. The capacities of HO• generated by different catalysts are consistent with the degradative efficiency tendency of MB, and hence the involvement of HO• in oxidizing MB is affirmed.

**Figure 7:**
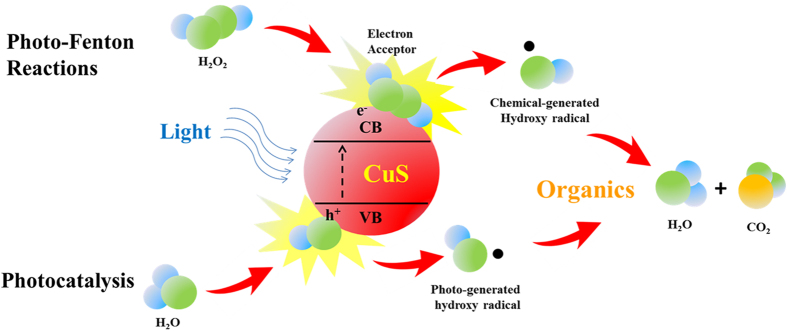
The photo degradation mechanism of the CuS catalysts.

Based on the above, the mechanism of the MB degeneration can be summarized by two pathways (Fig. 7). Chemical generated hydroxyl radicals can be produced from the reaction between the np-CuS and H_2_O_2_ via Fenton-like reactions. The Cu^+^ in the CuS could react with H_2_O_2_ to generate hydroxyl radicals, and further improve the photocatalytic property. On the other hand, photogenerated electron on the CuS can transfer from the valence band to the conduction band, and accordingly, a photogenerated hole can be formed. The photogenerated hydroxyl radicals can be produced by the reaction between the photogenerated hole and H_2_O. H_2_O_2_ is an efficient electron acceptor, the H_2_O_2_ can promote the separation of the photogenerated electron and hole, and more photogenerated hydroxyl radicals can be generated in the presence of H_2_O_2_. The MB can react with the hydroxyl radicals both generated from Fenton-like reactions and photocatalysis and finally be oxidized.

**Figure 8:**
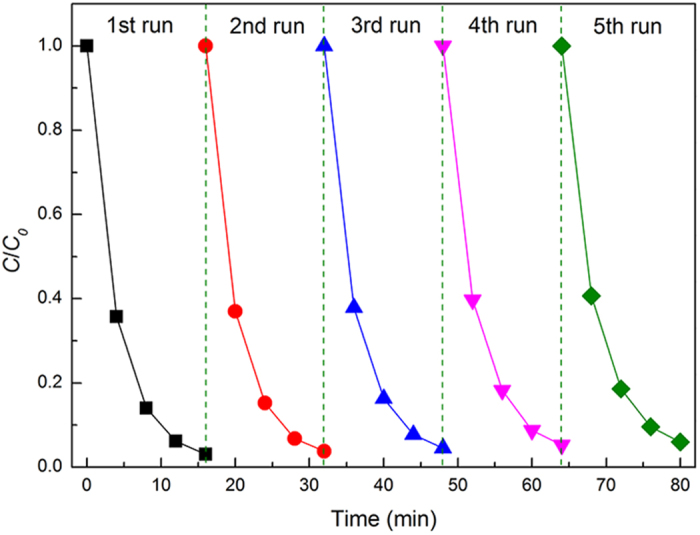
Time course of photocatalytic MB-degradation over nanoporous CuS.

For good photocatalysts, both activity and recyclability should be given consideration to characterize the photocatalytic performance. Figure 8 shows the reuse stability of the nanoporous CuS. No significant decrease was observed in photodegradation activity after five consecutive cycles. The degradation rate of MB after five cycles is still above 90%, indicating good recyclability. To further demonstrate the stability of the nanoporous CuS catalyst, SEM image of the CuS catalyst after five-cycle photocatalytic test is shown in Fig. S6, It is clearly that the CuS catalyst maintained the bicontinuous nanostructure after the photocatalytic test indicating good stability and recyclability of the nanoporous CuS catalyst.

## Conclusion

In summary, a novel np-CuS photocatlyst is synthesized by chemical dealloying TiCu amorphous alloy. The morphologies of the np-CuS catalyst can be controlled by the composition of the original amorphous alloys. The formation mechanism of the np-CuS involves three steps: (1) formation of the porous Cu_2_S nanostructure; (2) vulcanization of the Cu_2_S; (3) ripening of the np-CuS. The np-CuS exhibits excellent photocatalytic properties with the assistance of H_2_O_2_. In the solutions with the common test concentration of 10 mg L^−1^, MB, MO and RhB were degraded by the np-CuS with H_2_O_2_ very quickly. Especially MB solution with the concentration of 10 mg L^−1^ was degraded completely within 40 s. In high concentration MB solution (1000 mg L^−1^), 98% MB was degeneratied within 16 minutes. The excellent photocatalytic property of np-CuS is owing to the synergistic effect of np-CuS and H_2_O_2_. The np-CuS exhibits high specific surface area, high adsorption capacity and low recombination of the photo generated electrons and holes due to its bicontinuous nanostructure. The Cu^+^ in the CuS can react with H_2_O_2_ to generate hydroxyl radicals and further improve the photocatalytic property. The MB degradation mechanism can be ascribed to the photogenerated holes from the CuS and hydroxide radicals in the presence of CuS and H_2_O_2_. The hydroxyl radicals generated by two pathways made contributions to the oxidation of the dye molecules. The np-CuS exhibits good recyclability, the catalytic efficiency of the np-CuS maintained 90% after five cycles.

## Methods

### Materials

All the chemicals were obtained commercially without further purification.

### Synthesis of CuS catalysts

The CuS catalysts were prepared by a chemical dealloying method. Ti-Cu amorphous ribbons with different composition were prepared using a melt-spinning method in our previous work^40^. Pure Ti and Cu were arc melted into alloy ingots three times in an argon atmosphere. Then the alloy ingots were remelted in quartz tubes and ejected onto a Cu wheel rotating for rapid solidification to form the amorphous Ti-Cu alloys. The sulfuric acid solution with a concentration of 15 M was used as dealloying solutions. The amorphous alloy ribbons were cut into 4 cm in length and then immersed into the dealloying solutions in the reaction kettle. The reaction temperature and time were 363 K and 2 d, respectively. After the dealloying process, the products were washed in deionized water and absolute ethyl alcohol in sequence, then dried in drying oven in 303 K for 10 h. The products prepared by Ti_50_Cu_50_, Ti_40_Cu_60_, Ti_30_Cu_70_ and Ti_20_Cu_80_ were named as Sample 1, 2, 3 and 4, respectively.

### Characterizations

The morphology of the as-prepared sample was examined with a scanning electron microscope (SEM, Hitachi S4800) and transmission electron microscope (TEM, Jeol 2010). The acceleration voltage and working distance in the SEM test was 5 kV and 8 mm, respectively. X-Ray Diffraction (XRD, Bruker D8 instrument) X-ray photoelectron spectroscopy (XPS, PHL1600ESCA) and energy dispersive spectrometer (EDS, Genesis XM2) were used to determine the phase and composition. The specific surface area and pore size distribution were tested by nitrogen adsorption/desorption isotherms via a autosorb iQ instrument (Quantachrome U.S.) at 353 K. Photoluminescence (PL) measurements were performed on a spectrofluorometer (Horiba Jobin Yvon Fluorolog 3) at room temperature.

### Photocatalytic test

The photocatalytic activities of the catalysts were evaluated by the photodegeneration of aqueous MB, MhB and MO at ambient temperature. The light source was a 500 W Xe lamp, the distance between the lamp and the solution was about 10 cm and the illumination intensity was 0.01 W cm^−2^. For low dye concentration solution (for MB, MhB and MO, 10 mg L^−1^), 10 mg photocatalyst was added into 6 ml dye solution, and then 2 ml H_2_O_2_ solution (30 wt.%) was subsequently added into the mixed solution even without the adsorption/desorption equilibrium before the irradiation. The tests were ended when the dye was completely degenerated. For the high MB concentration solution (1000 mg L^−1^), 10 mg photocatalyst was dispersed in 6 mL MB solution. Prior to irradiation, the mixture was magnetically stirred in the dark for 40 min to establish an adsorption/desorption equilibrium, and then 2 ml H_2_O_2_ solution (30 wt.%) was added into the test solution. For the condition 5, N_2_ was injected into the MB solution before the addition of H_2_O_2_ to remove the dissolved O_2_. At given time intervals, 0.1 ml of the suspension was withdrawn and diluted in 2.9 ml H_2_O. After that, the diluted suspension was centrifuged to remove the remaining catalyst. The procedure of the recycle tests is same as the photodegradation experiments. The concentration of all dyes was monitored by a UV-vis spectroscopy (Shimadzu UV-2700). The amount of OH**·** radicals produced by the as-prepared materials was measured according to the ref. 22. Saturated benzoic acid (BA) solution was used as chemical probe to measure the time-dependent OH**·** radical concentration in H_2_O_2_ assisted Fenton reactions. It is reported that per mole p-hydroxybenzoic acid (p-HBA) was produced quantitatively by reacting 5.87 ± 0.18 moles OH**·** with BA. The p-HBA was quantitated by high performance liquid chromatography using a Agilent 1260 liquid chromatograph equipped with the Eclipse Plus C18 column (5 μm particle size, 15 cm length × 4.6 mm).

## Additional Information

**How to cite this article**: Xu, W. *et al.* Nanoporous CuS with excellent photocatalytic property. *Sci. Rep.* **5**, 18125; doi: 10.1038/srep18125 (2015).

## Supplementary Material

Supplementary Information
