# Supplementary material for: Nanoporous CuS with excellent photocatalytic property
Source: Sci Rep. 2015 Dec 9;5:18125. doi: 10.1038/srep18125 (PMC4673457; doi:10.1038/srep18125)
Supplement: Supplementary Information [file srep18125-s1.doc]

Nanoporous CuS with excellent photocatalytic property

Wence Xu^a^, Shengli Zhua, b, c, [[1]](#footnote-2), Yanqin Liang^a,b^, Zhaoyang Li^a,b^, Zhenduo Cui^a^, Xianjin Yang^a,b, 2^, Akihisa Inoue^a^

^a^ School of Materials Science and Engineering, Tianjin University，Tianjin, 300072, China

b Tianjin Key Laboratory of Composite and Functional Materials，Tianjin, 300072, China

c Key Laboratory of Advanced Ceramics and Mchining Technology, Ministry of Education, Tianjin, 300072, China


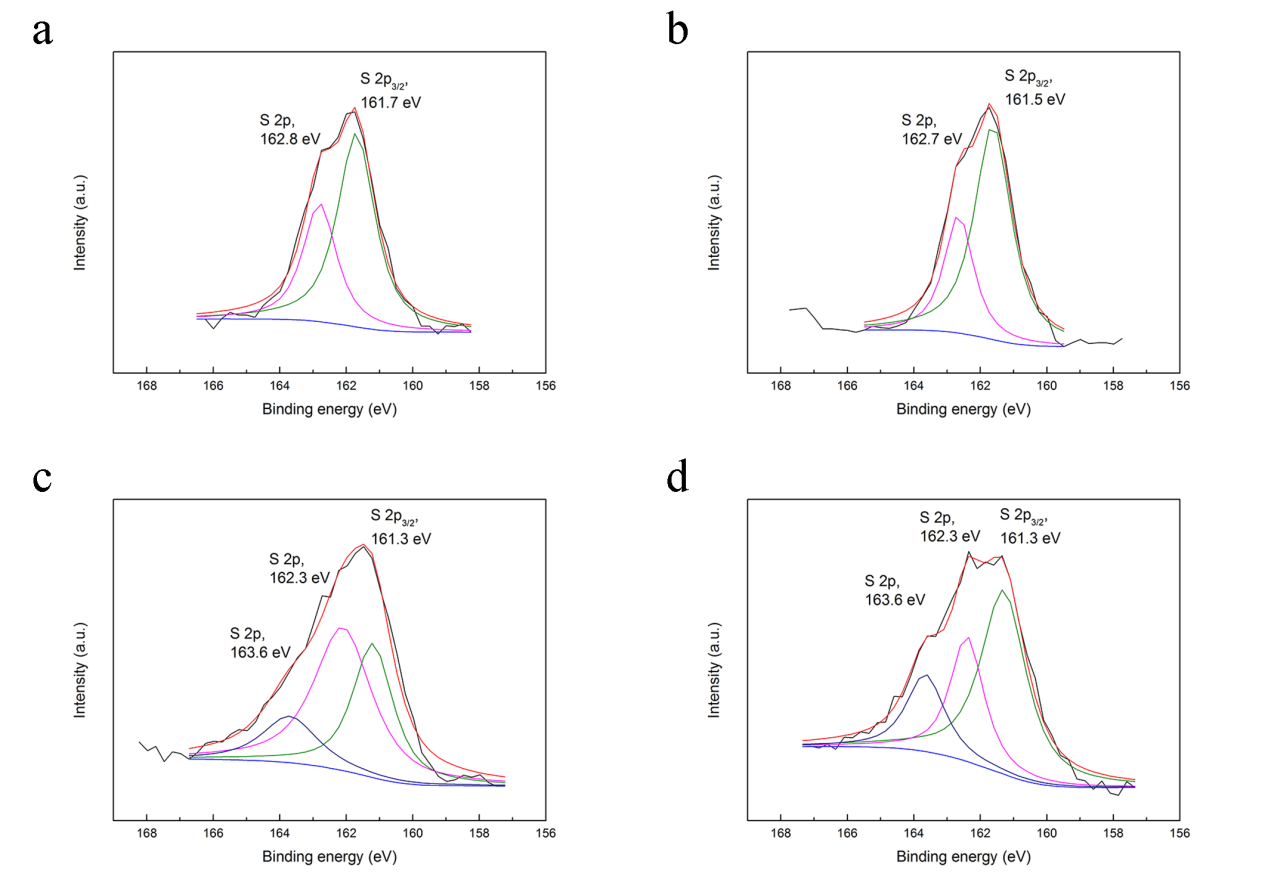


**Figure S1 |** Fitted XPS spectrum of S2p for the nanoporous CuS prepared by Ti_30_Cu_70_ amorphous alloy for (a) 5 min, (b) 1 h, (c) 16 h and (d) 2d.


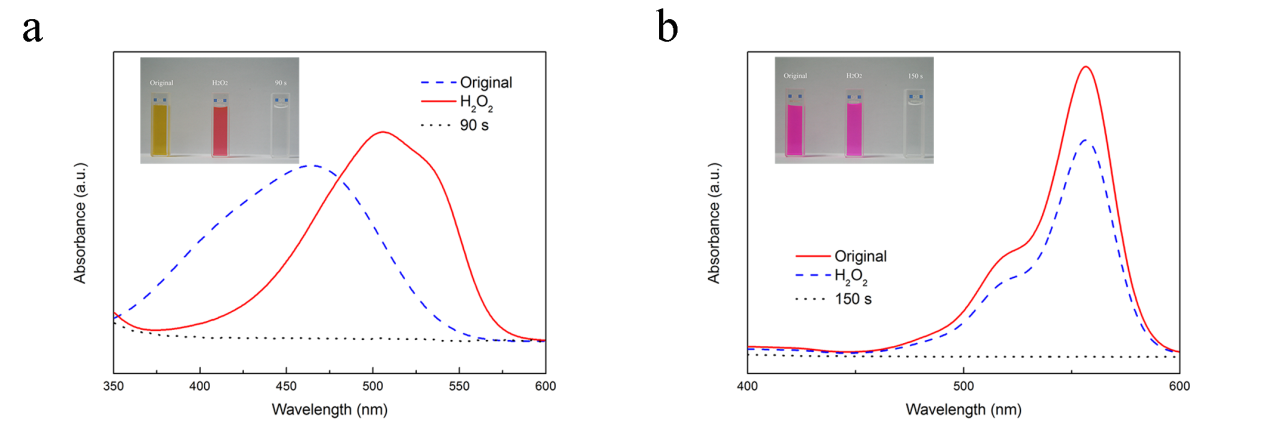


**Figure S2 |** Photodegradation curves of nanoporous CuS towards 10 mg L^-1^ methyl orange (a) and rhodamine B (b), the inset in (a) and (b) are the optical photos of the methyl orange and rhodamine B, respectively.


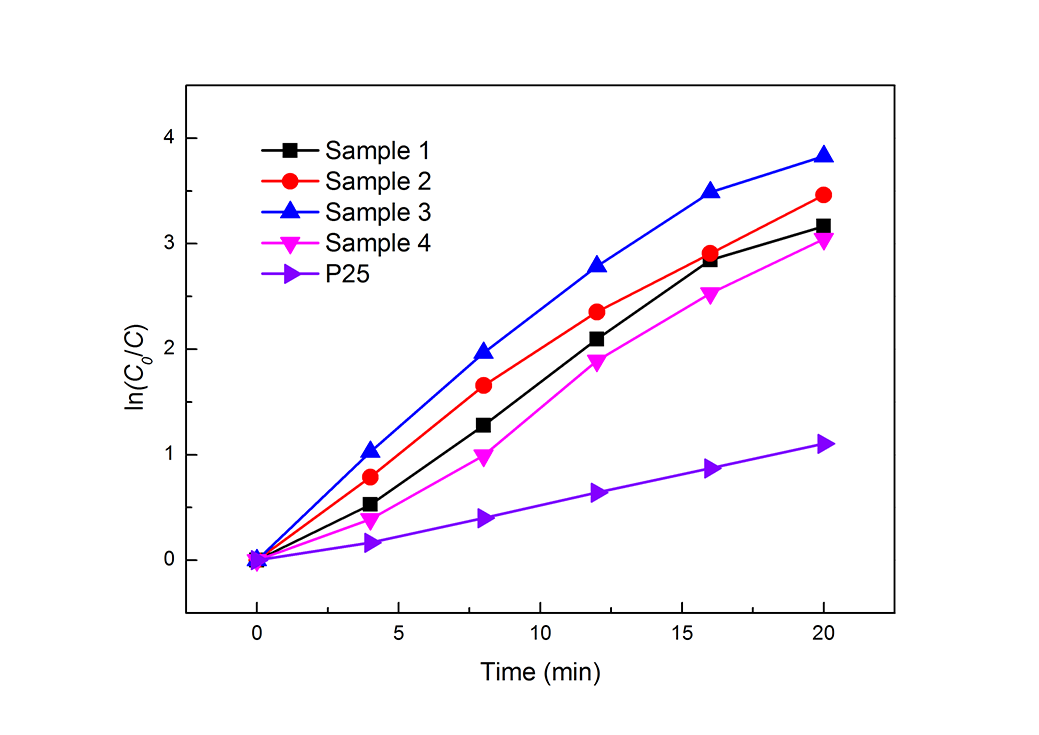


**Figure S3 |** Kinetic analysis of imidacloprid degradation, the plot of ln(C_0_/C) versus reaction time.


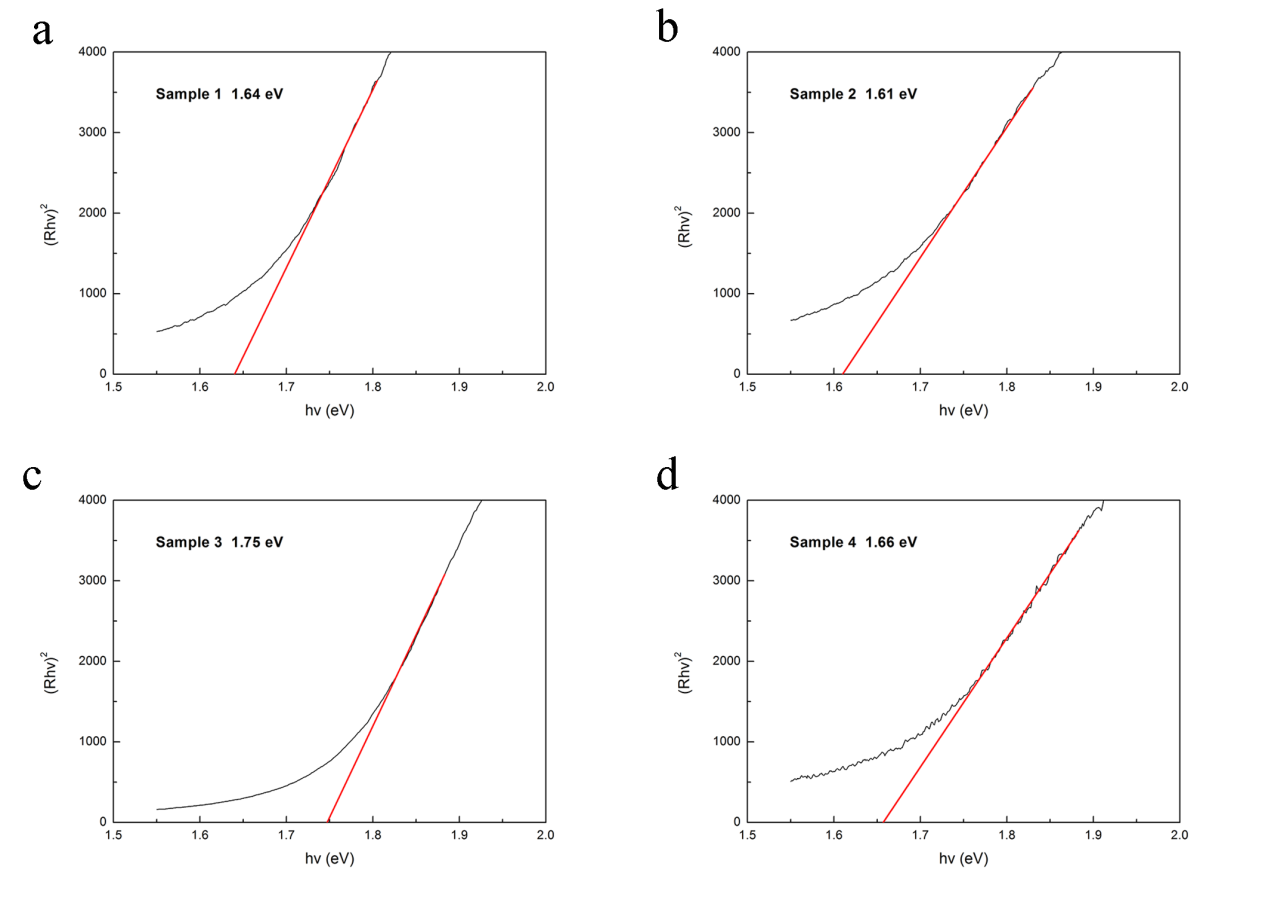


**Figure S4 |** Bandgap energy of the CuS catalysts prepared by Ti_50_Cu_50_ (a), Ti_40_Cu_60_ (b), Ti_30_Cu_70_ (c) and Ti_20_Cu_80_ (d) via Kubelka-Munk method.


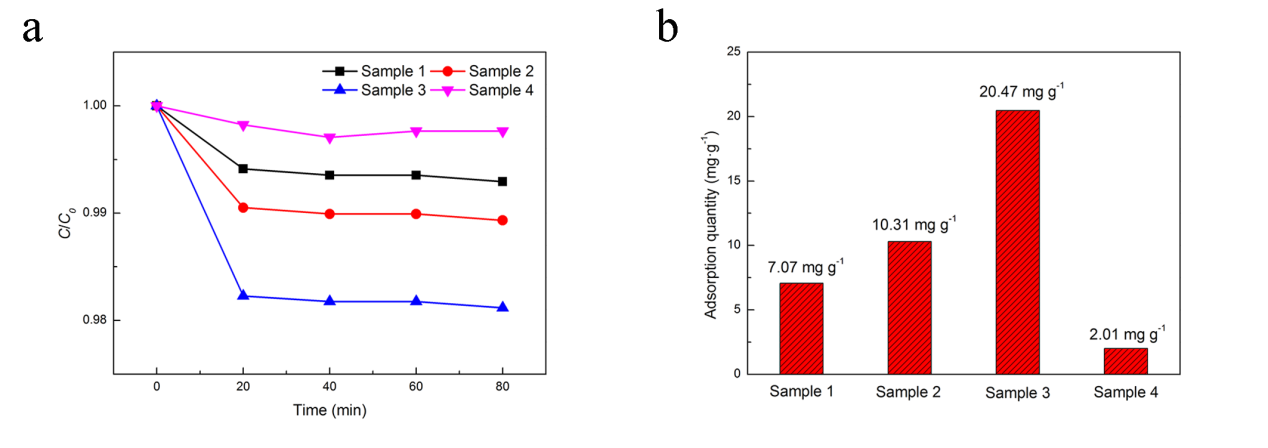


**Figure S5 |** (a) Adsorbent processes of the as-prepared CuS catalysts and (b) corresponding adsorbing capacities of these samples. The concentration of the MB solution is 1000 mg L^-1^.


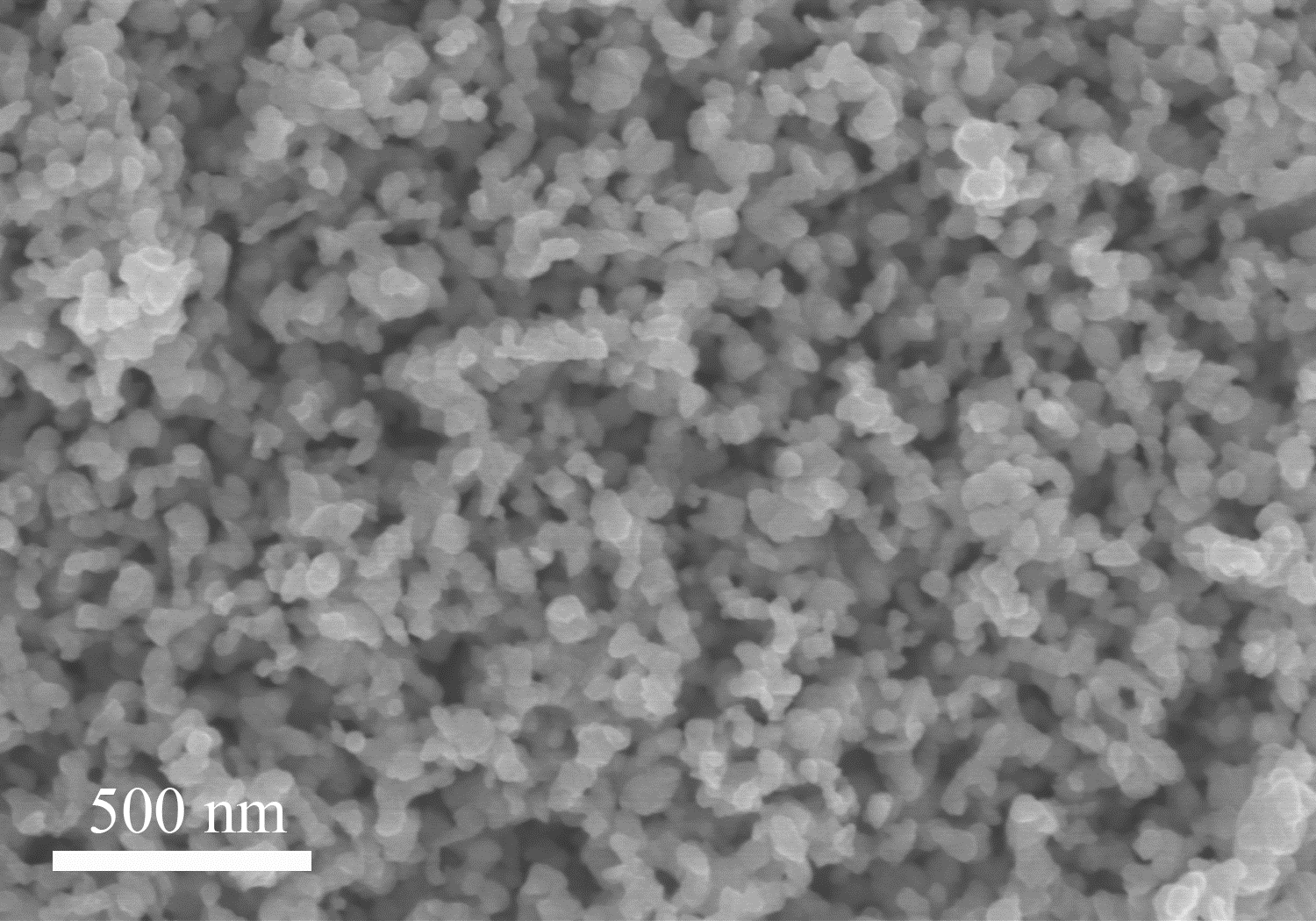


**Figure. S6 |** SEM image of the nanoporous CuS catalyst after five-cycle photoacatalytic test.

**Table S1.** Degradation rate constants of MB with different catalysts using Langmuir–Hinshelwood equation.

| Photocatalysts | *k* (min^-1^) | Raction Solution | References |
| --- | --- | --- | --- |
| Rutile TiO_2_/GQD | 0.0576 | 2×10^-5^ M L^-1^ MB | [1] |
| Ag_2_CrO_4_-GO | 0.28 | 1×10^-5^ M L^-1^ MB | [2] |
| CuS | 0.314 | 10 mg L^-1^ MB + 2.44 M H_2_O_2_ | [3] |
| Cu_2_O-Au | 0.00097 | 1×10^-5^ M L^-1^ MB | [4] |
| Cu_2_O | 0.00428 | 500 mg L^-1^ MB + 10 μL H_2_O_2_ | [5] |
| Sample 1 | 0.181 | 2.7×10^-3^ M L^-1^ MB + 2.44 M H_2_O_2_ | This paper |
| Sample 2 | 0.198 | 2.7×10^-3^ M L^-1^ MB + 2.44 M H_2_O_2_ | This paper |
| Sample 3 | 0.232 | 2.7×10^-3^ M L^-1^ MB + 2.44 M H_2_O_2_ | This paper |
| Sample 4 | 0.161 | 2.7×10^-3^ M L^-1^ MB + 2.44 M H_2_O_2_ | This paper |
| P25 | 0.056 | 2.7×10^-3^ M L^-1^ MB + 2.44 M H_2_O_2_ | This paper |

**Reference**

1. Zhuo, S., Shao, M. & S. T. Lee, Upconversion and Downconversion Fluorescent Graphene Quantum Dots: Ultrasonic Preparation and Photocatalysis. *Acs Nano* **6**, 1059-1064 (2012).

2. Xu, D., Cheng, B., Cao, S. & Yu, J. Enhanced photocatalytic activity and stability of Z-scheme Ag2CrO4-GO composite photocatalysts for organic pollutant degradation. *Appl. Catal. B Environ.* **164**, 380-388 (2015).

3. Mi, L. et al. Tunable properties induced by ion exchange in multilayer intertwined CuS microflowers with hierarchal structures. *Nanoscale* **5**, 6589-6598 (2013).

4. Pan, Y. et al. Plasmon-Enhanced Photocatalytic Properties of Cu2O Nanowire-Au Nanoparticle Assemblies. *Langmuir* **28**, 12304-12310 (2012).

5. Kumar, B., Saha, S., Ganguly, A. & A. K. Ganguli, A facile low temperature (350 degrees C) synthesis of Cu2O nanoparticles and their electrocatalytic and photocatalytic properties. *Rsc Adv.* **4**, 12043-12049 (2014).

1. Corresponding author. Tel: +86 22 27402494; Fax: +86 22 27404724; E-mail: [slzhu@tju.edu.cn](mailto:slzhu@tju.edu.cn)

   2 Corresponding author. xjyang@tju.edu.cn [↑](#footnote-ref-2)
